# Prior Knowledge, Episodic Control and Theory of Mind in Autism: Toward an Integrative Account of Social Cognition

**DOI:** 10.3389/fpsyg.2018.00752

**Published:** 2018-09-05

**Authors:** Tiziana Zalla, Joanna Korman

**Affiliations:** ^1^Institut Jean Nicod, CNRS UMR 8129, Institut d’Etude de la Cognition, École Normale Supérieure and PSL Research University, Paris, France; ^2^Navy Center for Applied Research in Artificial Intelligence, United States Naval Research Laboratory, Washington, DC, United States

**Keywords:** theory of mind, prior knowledge, cognitive control, episodic buffer, social priors, compensatory processes

## Abstract

Over the last 30 years, research has explored theory of mind (ToM), the ability to attribute mental states to oneself and to others. Work on ToM in typical and atypical populations has shed light on the neurocognitive mechanisms underlying social understanding and interaction. The ToM hypothesis has long been regarded as one comprehensive explanation of the severe cognitive and behavioral impairments encountered by individuals with autism. However, high-functioning individuals can often pass both first-order and second-order false belief tasks using cognitive compensation strategies. To provide more sensitive measures of mental state attribution abilities, researchers have introduced more difficult, “advanced” theory of mind tasks. In this article, we argue that in attempting to bypass compensation strategies, these new advanced ToM tasks, such as the Faux Pas and the Strange Stories tasks, impose cognitive demands beyond those specific to the domain of ToM. We then provide an integrative account of social deficits in autism that takes into account several distinct components of mental state understanding, including both general cognitive capacities and processes specific to ToM. We argue that a number of related cognitive abilities, including episodic cognitive control and inferencing from prior knowledge, are necessary to understand how both people with autism and typical development navigate challenging, real-life social situations.

## Introduction

Autism spectrum disorder (ASD) is characterized by deficits in social interaction and communication. Over the last 30 years, one prominent explanation for these deficits has been impairments in theory of mind (ToM), also known as mentalizing: the ability to attribute mental states, such as thoughts, intentions, beliefs, and desires, to oneself and to others (Baron-Cohen et al., 1985; Leslie, 1987; Frith, 1989; Frith et al., 1991; Hamilton, 2009). ToM is often studied using a suite of closely related tasks; most notably, the false belief task and its variants (Happé et al., 2017). The finding that young children with autism struggle on false belief tasks while typically developing children pass them is well-established in many studies (Baron-Cohen et al., 1985; Happé, 1995). There has emerged a clear and robust dissociation between typically developing and autistic children on the construct of ToM. Not only do typically developing children have generally intact ToM while children with autism tend to exhibit severe difficulties; even developmentally disabled, non-autistic children with considerably lower general intelligence than children without delay can be distinguished from children with autism on ToM measures (Baron-Cohen et al., 1985, Happé, 1995). ToM deficits have thus been thought to be a distinguishing feature of autism that also has the potential to explain many social impairments. However, the performance of adolescents and adults with high-functioning autism spectrum disorder (HFASD) – those with normal intellectual functioning – on these tasks poses a conundrum. HFASD individuals appear to have a late-acquired ToM capacity: they can often pass both first-order and second-order false belief tasks (Bowler, 1992, Happé, 1995; Dahlgren and Trillingsgaard, 1996). Yet, researchers have argued that, rather than a late-acquired ToM competence, different types of neurocognitive adaptive and compensatory strategies – distinct from the mental-state specific ToM capacity, *per se* – are responsible for these successes (see Livingston and Happé, 2017). To provide more sensitive measures of ToM abilities, researchers have thus introduced more difficult, “advanced" ToM tasks, which are meant to prevent the use of compensation strategies, and thus demonstrate that adolescents and adults with autism spectrum disorder (ASD) lack or have a diminished ToM (Rajendran and Mitchell, 2007).

In the present article, however, we argue that such compensation strategies are not merely a nuisance to be worked around. Indeed, the term “compensation,” which suggests a papering over a missing ToM capacity via a distinct route largely irrelevant to the capacity in question, is something of a misnomer, since domain-general cognition is also a crucial part of ToM functioning in typical development (Apperly, 2010; Korman et al., 2015). To explore the role of such capacities in ASD social functioning, we highlight the general cognitive demands made by conventional ToM tasks, as well as accounts of ASD individuals’ general cognitive capacities in a range of domains, including inferences from general event schemas and prior knowledge, episodic memory, and executive functions. By considering both the successes and limitations of general cognitive compensation strategies in HFASD, we propose an integrative account of social difficulties in autism. Such an account would explain performance not only on social tasks intended to capture the construct of ToM, but also in tasks that more closely mimic the demands of real-life, ecological social interaction.

In many challenging, real-world ToM problems, general cognitive capacities play a crucial role. For example, in order make use of incoming social information in online inferences about others’ mental states, people rely on the situation at hand to activate relevant social information in episodic memory (Higgins, 1996). Consider a situation in which a familiar customer walks into an electronics store wanting to return a defective speaker system. In such a situation, the clerk who helped him select the system on his previous visit will facilitate a more successful interaction if she possesses not only a general conceptual ability to represent the mental states of other people, but remembers crucial details of the interaction: for example, that this particular customer had searched for weeks for this particular system, and he was insistent that the clerk vouch for their quality (that is, he had a *desire* for high quality). Without these memories, and, crucially, the ability to activate them when the customer walks in demanding a refund, the clerk may simply default to company policy that “all sales are final.” Irate that the product was not as promised and disappointed in the clerk’s lack of regard for his time and money, the customer may leave in a huff. In such a situation, the mere capacity for abstract mental state understanding is not sufficient. Only the clerk’s ability to recall the specific contents of the customer’s previous mental states relative to the situation, and to draw further inferences about the customer’s needs and wishes based on these social and contextual memories, allows her to respond in the most socially beneficial way. Here, we argue that an integrative approach to ToM – one that takes into account capacities specific to mental state understanding, as well as the relationship between these capacities and general cognitive abilities – is necessary to understand how typically developing adults navigate challenging social situations.

In setting forth such an approach, in the first section, we begin with a discussion of advanced ToM tasks and HFASD adults’ performance. In the next section, we discuss the new demands on general knowledge introduced by advanced ToM tasks beyond those of the false belief tasks. We then set forth an application of recent theories of general prior knowledge, such as the hypo-priors hypothesis (Pellicano and Burr, 2012), to the case of social and intentional reasoning. Finally, we explore how deficits in cognitive control and episodic memory may explain ToM deficits. Specifically, we argue that deficits in social reasoning and interaction may be explained by disruptions in the episodic control system and episodic buffer, which in TD individuals, serves to retrieve social and episodic autobiographical knowledge from long-term memory and maintain it in working memory.

## Thirty Years of ToM Studies: From False Belief to the Advanced ToM Tasks

The false belief task has been widely used in studies of both autism and typical development (TD). In one classic version (Baron-Cohen et al., 1985), one character places an object in a particular location (e.g., a basket). That character then leaves the scene and a second character moves the object to a different location (e.g., a box). The first character then returns to the scene, and the participant is asked to report (in one version) where the second character *believes* the item to be. If “theory of mind” is defined as the ability to attribute beliefs to another person, then the concept under study (belief) is clear enough in this task.

One explanation commonly cited for ASD adults’ competence on first- (as in the task above) and second-order false-belief tasks is that they engage in “compensation,” or “hacking out” strategies (Eisenmajer and Prior, 1991; Frith et al., 1991; Happé, 1995). In this context, this term refers to general cognitive strategies used by ASD individuals to pass false belief tasks, which are thought to be distinct from the mental-state-specific ToM abilities possessed by typically developing individuals. For example, instead of engaging in an inference of a particular character’s mental state, individuals on the autism spectrum might pass false belief tasks by using a rule-based strategy. They might come to learn a rule, such as ‘perception leads to knowledge,’ and they could thus conclude that whenever a character lacks perception of a particular fact, that character will lack knowledge of that fact. One major aim of advanced ToM tasks was to develop a test for which such compensation strategies would not be easily employed, particularly for drawing belief inferences. By embedding the need for a mental state inference in a richer, somewhat more realistic social context, researchers hoped to prevent ASD participants from engaging in such strategies.

Two of the most common advanced ToM tasks developed for this purpose are the “Strange Stories” and “Faux Pas” tasks. The original Strange Stories task (Happé, 1994) depicts utterances with features such as sarcasm, persuasion, double bluff, lying, misunderstanding, and forgetting. In one story depicting sarcasm, for example, a girl named Ann is watching TV and doesn’t look up or say “thank you” when her mother brings her favorite meal. Ann’s mother becomes cross and says, “Well that’s very nice, isn’t it! That’s what I call politeness!” Participants’ task is to evaluate whether what Ann’s mother says is true, and to explain why she says this. The Faux Pas task (Baron-Cohen et al., 1999) also depicts an utterance, but one that is well-meaning, intended literally, and that has an unanticipated effect on the hearer. For example, when one woman moves into a new apartment and buys new curtains, her friend comes over, looks at the curtains, and remarks that she likes the apartment, but that, “the curtains are awful! I hope you’re going to get new ones!” In this task, the participant must recognize that the utterance, while intentional, is offensive and misdirected, and leads to an awkward situation in which the hearer unintentionally reveals a hidden belief.

While these novel tasks may be successful in preventing ASD adolescents and adults from engaging in compensation strategies, they also introduce new conceptual and processing demands beyond those required for the false belief tasks. These demands include (1) those specific to ToM concepts and (2) general demands on inferential capabilities. In the following two sections, we discuss each of these demands in turn.

## Challenges Beyond Compensation Strategies: Introducing New Conceptual and Inferential Demands

Researchers have demonstrated that the ToM capacity involves a suite of concepts uniquely suited for understanding other minds. However, not all ToM concepts are the same: for example, desire is acquired before belief (Wellman and Woolley, 1990; Bartsch and Wellman, 1995), and some more advanced concepts continue to develop well into adolescence. A change in the concepts measured from the false belief task may thus constitute a significant change in task demands. Indeed, although the distinct types of utterances depicted in the Strange Stories and Faux Pas tasks all require belief understanding, they also require a mastery of several distinct concepts beyond belief, some of which are not mastered by typically developing children until long after the preschool years, if ever (Filippova and Astington, 2008). For example, sarcasm and irony, which are depicted in the strange stories, are complex feats of social communication that, even for typically developing (TD) individuals, continue to develop well through the elementary and middle school years. To accurately interpret a sarcastic statement, an individual who observes the interaction has to understand that (1) Anne’s mother’s utterance is not literally true (Ann is not really acting politely); (2) Anne’s mother is aware of the utterance’s untruth (She believes that Ann’s behavior is impolite), (3) Ann’s mother intends to communicate to Ann that she believes the utterance is untrue, and, crucially, (4) Ann’s mother chooses an ironic, rather than a direct statement, in order to communicate particular attitude (i.e., she believes that Ann should really know better than to behave in such an impolite way). As Filippova and Astington (2008) and Zalla et al. (2014) demonstrated, not even TD adults are at ceiling in accurately interpreting the pragmatic force of the speaker’s attitude. Thus, a comparison between TD and ASD adults on irony understanding may be a specious one. While a task like the strange stories assumes that irony is an ‘advanced’ competency possessed by all TD adults, this competency may be so advanced that its mastery represents an idealized, rather than an actual, portrayal of social cognition in TD adults.

The Faux Pas task has similar limitations, requiring the participant to recognize not only the speaker’s actual communicative intent, but also the speaker’s later feeling of awkwardness or embarrassment about her remark when she realizes the unintended thoughts and feelings it has likely evoked in the listener. In both tasks, the observer has to interpret not only a second-order belief on the part of the speaker, but a second-order belief that is in dynamic interaction with the beliefs of the listener (Carpendale and Chandler, 1996). Furthermore, each agent in the story forms his or her beliefs based on access to different background information.

These are challenging conceptual demands, perhaps even for older TD children and adults. Indeed, TD adults do not perform at ceiling on a number of questions on the faux pas task (Zalla et al., 2009). Thus, the mere fact that TD adolescents and adults often perform better than ASD individuals on the aforementioned “advanced” ToM tasks is not definitive evidence for differences in ToM concepts. In order to demonstrate such differences, the precise concepts being measured need to be specified more clearly.

An appeal to ToM concepts is, of course, a common way of explaining ASD individuals’ deficits on such tasks, but there are also alternative explanations. One is that the very same novel social situations that *prevent* participants from appealing to simple compensatory rules about visual access to make a ToM inference are also responsible for introducing novel inferential demands – demands that are distinct from ToM reasoning *per se*, yet still important to its execution.

Understanding social situations like those depicted in the advanced ToM task draws, not only on ToM concepts, or on specific processes associated with ToM, such as simulation, but also, quite crucially, on inference processes that draw on general semantic knowledge, situational script knowledge or episodic autobiographical memory. Thus, as we and our collaborators have argued previously, a general cognitive explanation for failure on advanced ToM tasks – albeit one distinct from a rule-based appeal to visual access – is actually quite difficult to rule out (Korman et al., 2017; Korman et al., unpublished).

Consider the Faux Pas story, also described in Korman et al. (2017), in which one woman’s friend inadvertently insults her curtains. Although the story states outright that the woman has just moved into her new apartment, it does not explicitly state that her friend has never been there. Thus, it remains possible that the friend *has* been there before, and *knows* that the curtains do not originate from the previous owner. On this interpretation of the scenario, the woman’s friend has a *true* belief about the origin of her curtains, and her statement about the curtains can be seen as deliberately insulting. Of course, it may seem obvious to a TD individual that the friend is coming to the apartment for the first time; this seems a reasonable inference to make from the information provided. However, it is precisely this type of seemingly obvious inference – people who rent new apartments do not generally have all of their friends over immediately upon moving – that ASD individuals may struggle with. Importantly, it is these types of inferences, which are drawn from general and contextual knowledge, and do not even directly involve mental states, which may ultimately be consequential for mental state inference (see Korman et al., 2017). Without realizing that the woman’s friend has not been to the apartment before, it is more difficult to reach the inference that she has a false belief about the source of the woman’s curtains.

A growing literature documents general inferential difficulties in individuals on the autism spectrum. For example, Bodner et al. (2015) have noted inference deficits in ASD individuals, not only for emotion-related stories, but for complex physical stories designed to be at a comparable level of difficulty to social stories. These difficulties suggest that, beyond a specific social deficit, ASD individuals may have a more general deficit in the ability to apply existing world knowledge to novel contexts. Indeed, they often fail to represent variable aspects of common social events (Loth et al., 2008, 2011; Zalla et al., 2010) and to spontaneously attend to situational information that is most relevant to a high-level event schema (Zalla et al., 2006; Loth et al., 2011). Zalla et al. (2006) also showed that children with ASD had difficulties in constructing sequences of common social goals, also known as “scripts” (Schank and Abelson, 1977). “Scripts” represent the hierarchical and temporal relationship among the single events constituting knowledge of common social events. For example, TD individuals are able to infer that, after moving to a new apartment, a person first normally arranges the furniture and unpacks boxes, and only afterward invites guests over. TD individuals also recognize that performing these actions in the opposite order would be atypical. ASD individuals’ struggle with drawing inferences based on script and event knowledge may, by itself, account for some of their social difficulties, including their poor performance on advanced ToM tasks. If ASD individuals are less able to draw on their past experience and script knowledge to make simple inferences in a range of social and non-social situations, they may never reach the basic conclusions required to apply ToM concepts in the first place.

In recent studies with collaborators, we have tested these hypotheses of increased conceptual and inferential demands (Korman et al., 2017; Korman et al., unpublished). Using a modified version of the Faux Pas task, we have shown that HFASD individuals are capable of adequately fulfilling the new conceptual demands of the Faux Pas task. HFASD individuals are able to understand the basic intentions behind the speaker’s action and the awkwardness experienced by the speaker, both in the presence and absence of explicit information about the her/his positive desire and false belief. In addition, surprisingly, presenting new Faux Pas stories containing key inferential background information appears to lead to an improvement in overall performance on the task relative to previous studies that lack such information. This finding, though preliminary, suggests that ASD individuals’ impairment in generating correct mental states could be due to an inability to draw appropriate general inferences from background knowledge, rather than an inability to infer mental states, *per se*.

## Social Priors in Autism: Intention Inference and Social Learning

In recent years, cognitive theories of autism have sought to go beyond merely explaining deficits in *social* inference in autism. In particular, Bayesian models of cognition have ventured to explain other characteristic features of ASD, such as enhanced perceptual functioning, an experience of sensory overload, and hypersensitivity to stimuli. Two theories have provided accounts of these features. One proposes that they may be explained by a diminished influence of *top-down* prior expectations (“hypo-priors) in drawing inferences from the world (Pellicano and Burr, 2012), while another theory holds that these symptoms are best explained by *bottom-up* functioning and greater weighting of sensory information (“enhanced sensory precision”) in drawing such inferences (Lawson et al., 2014). Regardless of which of these hypotheses ultimately proves most fruitful, this general approach characterizes two key inferential capacities that are crucial to ToM inferences in typical development. In particular, it specifies how a social perceiver can draw inferences from behavior in real time. As an agent’s behavior unfolds, the brain of the social perceiver draws inferences by using two distinctive types of information. First, the social perceiver takes into account the sensory evidence conveyed by movement kinematics. Second, the social perceiver also assesses, based on her expectations, the intention which likely causes the observed action (Baker et al., 2006, 2009). Observers must rely more on their prior expectations as the reliability of sensory evidence decreases, and more on sensory inputs when priors are not available. Thus, in situations of noisy or incomplete sensory evidence, typically developing individuals tend to compensate for visual uncertainty by appealing to prior knowledge (Baker et al., 2009).

How are these two key inferential capacities and their interplay manifest in high-functioning ASD individuals? Pellicano and Burr (2012) have suggested the hypothesis that if prior expectations and knowledge from past experience are lacking or reduced in individuals with ASD, they may perceive the physical world more accurately than TD individuals, but possess an incomplete and fragmentary psychological understanding. Indeed, understanding the individual actions in an interactive context or predicting the outcomes of social interactions could be particularly difficult if the observer depends on perceptual evidence only, without being able to call on precise prior knowledge to contextualize other people’s behaviors. Because a single observable action (i.e., smiling) is in principle consistent with multiple underlying intentions (e.g., to communicate pleasure; to ironically communicate displeasure), it can be difficult to disambiguate social intentions on the basis of such observational data alone. Hence, prior knowledge is required to constrain the range of competing intentions that are potentially consistent with sensory information.

In a recent study, Chambon et al. (2017) used a Bayesian approach to investigate the ability of adults with ASD to infer non-social and social intentions. ASD and comparison participants were presented with a series of video clips showing an actor manipulating (either transporting or rotating) a non-meaningful object and asked to infer the actor’s intention, which was directed either at the object in a private context (non-social intention condition) or at a third party (social intention condition). The ability to predict the action of individuals acting in a social context requires the rapid analysis of visible sensorimotor information to extract information about hidden states, including the first-order mental states (i.e., beliefs, intentions) and second-order mental states (i.e., beliefs about beliefs) of multiple agents. However, given the complexity and the quantity of information to infer and hold in mind in a dynamic and rapidly unfolding scenario, social perceivers may often need to rely more on prior knowledge or other heuristics to form mental state attributions and select their own actions more efficiently. Prior knowledge of the world’s regularities, which allows disambiguation of the incoming sensory information, may thus play a crucial role in the top-down modulation of low level sensory processing. Such modulation would require retrieval of relevant social information from episodic autobiographical memory about the agent or script knowledge about a sequence of conventional actions.

In the study conducted by Chambon et al. (2017), the specific contributions of sensory evidence and prior expectations to mental state attribution and action selection were systematically manipulated across the social and non-social intention conditions. To do this, the experiments varied the amount of visuomotor information displayed by the action scene (i.e., the completeness of action sequences) and the probability of occurrence associated with two distinct candidate intentions (the agent’s intention to rotate versus transport a cube in the non-social condition, and to cooperate vs. defect in the social condition). Results revealed that prior information exerted a greater influence on the inference of social intentions than on non-social intentions in typically developing adults. In contrast, the effect of prior expectations was similar across the two conditions in ASD adults. Moreover, while the magnitude of the effect of prior knowledge was greater in the social than in the non-social task in the control group, prior knowledge exerted a similar influence on the ASD group’s accuracy in both tasks. Consistently, the influence of priors on participants’ responses in the social and non-social tasks was highly correlated in the ASD group, but uncorrelated in the control group. This finding suggests that, unlike in controls, in ASD, the same neurocognitive mechanism subserves social and non-social inferential processes. Importantly, rather than suggesting that ASD individuals have a generalized impairment in inferring other people’s intentions (i.e., a general decrease in performance for both social and non-social intentions), Chambon et al. (2017)’s study revealed a selective impairment in the inference of (‘tit-for-tat’) ‘reciprocating’ intentions in ASD. While the top-down influence of social-specific priors was markedly attenuated, bottom-up sensory processing was normal.

This study revealed that the ability to predict events may further vary depending on the type of intention to be inferred: action prediction relies more heavily on prior experience than on sensory evidence when inferring intentions in a social interactive context (i.e., a Tit-for-Tat mode of interaction between two individuals, wherein, for example, one individual cooperates in response to the others’ cooperation), while the role of prior knowledge is less prominent for intentions in a private context (i.e., directed at objects in isolation). Because of the reduced effect of social prior knowledge in individuals with ASD, “top-down” control mechanisms, operating on prior knowledge, fail to constrain sensory processing during social interaction. This interplay explains why ASD deficits are more pronounced in social-interactive situations, where priors are of particular importance. Chambon et al. (2017)’s study suggests that, compared to private intentions directed toward objects, social intentions may be uniquely difficult for ASD individuals. Thus, while in typically developing individuals, prior knowledge may come into play where there is a lack of sensory information and physical constraints, for individuals with ASD, the attenuated effect of prior knowledge has a negative impact on social understanding and behavior.

Interestingly, this study also revealed that, although participants with ASD did not exhibit spontaneous preference for TFT mode of reciprocation, they were able to acquire this *social* bias progressively, likely through a probabilistic learning mechanism which also operates for inferring private intentions from motor actions. Importantly, in addition to highlighting the close connections between ToM deficits and the lack of social priors, Chambon et al. (2017)’s findings also seem to suggest that ASD individuals are capable of using general cognitive strategies to achieve sophisticated social learning. That is, adaptive prior social biases held by TD individuals, such as the tendency to assume that a cooperative action by one agent will be reciprocated by his partner, might be taught to individuals with ASD. However, individuals with ASD showed a lack a spontaneous preference for the TFT mode of reciprocation. Thus, it is still up for debate whether they can consolidate and store social information acquired through general probabilistic learning mechanism. Indeed, it is possible that difficulties in recruiting prior knowledge, and their contributions to deficits in mental state ascription, are due to a broader deficit in ASD in consolidating episodic information from past experience in long-term memory.

Beyond mental state ascription, another area in which ASD individuals’ priors have been examined is in the domain of social reputation. Maurer et al. (2018) recently investigated the effect of prior reputational information and reciprocity in adults with and without ASD using a multi-round Trust Game (MTG). The participants played the role of investors and each counterpart was introduced to a player with a short biographical description about relevant personal events and professional achievements. Two ‘good’ counterparts were depicted as having committed praiseworthy actions (positive reputational prior) while the two ‘bad’ counterparts were depicted as having committed blameworthy actions (negative reputational prior). Crucially, during the MTG, one of the ‘good’ partners behaved in an individualist manner and one of the ‘bad’ partners behaved in a collaborative manner, so as to create two congruent and two incongruent profiles.

All participants were sensitive to the counterpart’s reputational priors and reciprocity, as they were influenced by the reputational priors in their initial decision to trust or distrust the counterpart, i.e., they were prone to invest more with counterparts with a positive reputation than with those with a negative reputation. Crucially, control (TD) participants utilized a TfT strategy, adapting their investment strategies mainly on the basis of the counterpart’s cooperative or individualist attitudes. Thus, for them, at the end of the game, the reputational prior was no longer highly influential, and their decision to trust or not trust was mainly affected by the counterpart’s direct reciprocity. Conversely, although ASD participants were sensitive to reciprocation, the reputational prior continued to exert a strong impact on their trust behavior: they were highly reluctant to reciprocate with participants with incongruous profiles. They never completely trusted the counterparts with a negative reputation, even if one of the two counterparts repeatedly showed collaborative behavior, and they continued to put more trust in the counterparts with a positive reputation, even when one of them adopted an uncooperative behavior.

Overall, these findings suggest that although preserved general learning abilities might sometimes sustain social learning, spontaneous and implicit social learning appears to be diminished in individuals with ASD. Because of the attenuated effect of social priors for reciprocating intentions, ASD individuals have difficulty predicting others’ behavior in an interactive context. In addition, when engaged in interactive exchange, even when they are provided with explicit prior knowledge for their inferences, they do so awkwardly or inflexibly, which results in a reduced sense of reciprocity. Thus, beyond an attenuated effect of priors, these results suggest that individuals with ASD have difficulties in flexibly integrating prior knowledge and feedback learning. These results suggest that inflexible social interaction and difficulties with social learning in ASD are explained by a disturbance in the Bayesian inferential mechanisms that adaptively regulate the interplay between prior expectations and sensory information.

So far, we have seen that a number of mechanisms related to prior knowledge may contribute to ASD adults’ difficulties with mental state inference. In static theory of mind tasks such as the Faux Pas task, ASD individuals may fail to draw on prior knowledge to make general inferences that are required before mental state inference can take place. In the case of reciprocating intentions, ASD individuals seem to exhibit a diminished ability to use prior knowledge both to predict other’s behavior and to respond adaptively during social exchanges. When provided with explicit prior knowledge, they encounter difficulties using this information flexibly and adaptively in an interactive context.

## The Integration Deficit Hypothesis

In addition to prior knowledge, a number of distinct sources of information inform social cognition. Achim et al. (2013) proposed a model in which four distinct sources of immediately obtainable information for ToM reasoning are identified: (i) perception about the agent (person or character) to whom the intentional state is attributed (ii) linguistic information about the agent, (iii) linguistic information about the environment and (iv) perceptual contextual information about the environment. Achim’s model also includes four sources of information in memory – that is, those that are derived from prior knowledge: (i) knowledge about a specific person, (ii) general knowledge about relatively unspecific people, (iii) knowledge about a specific context, (iv) knowledge about relatively unspecific contexts (i.e., prototypical or scripts).

Within this framework, difficulties with advanced ToM tasks or mental state ascription in real-life situations in individuals with ASD may reflect either the lack of immediate or stored sources of information or the inability to efficiently and automatically integrate information about mental states (i.e., knowledge about a specific person) with knowledge about the social situation (i.e., knowledge about a person’s behavior, the behavior’s broader context, or the behavior’s effect on others).

Empirical support for the integration hypothesis is provided by studies investigating the relationship between intentional judgment and moral reasoning in adults with ASD (Moran et al., 2011; Zalla and Leboyer, 2011; Zalla et al., 2011; Buon et al., 2013). For TD individuals, intention information is a crucial component of moral reasoning (Cushman, 2008; Malle et al., 2014). Zalla et al. (2011) reported that adults with HFASD have difficulty providing appropriate moral justifications because they fail to use multiple sources of information about the agent’s intentions, actions and affective states in conscious moral reasoning. Notably, while some ASD individuals have difficulty using intentional cues to correctly ascribe mental states, others struggle to use information about the agent’s intentions for moral reasoning even when the agent’s intention is correctly identified (Zalla and Leboyer, 2011; Zalla et al., 2011; Buon et al., 2013). Buon et al. (2013)’s study investigated the ability to assign moral responsibility and punishment in adults with ASD using non-verbal cartoons depicting intentional harm, accidental harm or a harm that was merely coincidental. Participants were asked to evaluate the agent’s intentional and causal roles in bringing about harm, his responsibility in the harmful situation, and the punishment the agent deserves for his action. Buon et al’s. (2013) study showed that, in their judgments of causality, adults with ASD failed to take into account the intentional state of the agent, and had difficulty reconciling information about the agent’s intentional states with the action’s outcome. That is, they tended to overstate the agent’s intention to do harm in the accidental condition and to understate the agent’s intention in the intentional condition. When asked to judge the agent’s responsibility and rate the punishment he deserved, individuals with ASD regarded the agent as being more responsible in the accidental and coincidental conditions than did control participants, and they punished him more severely than control participants in the accidental condition. In addition, even when they correctly attributed intentions and mental states to an agent, a subgroup of individuals with ASD failed to appeal to mental state information to justify their moral judgments (Buon et al., 2013). These findings support the hypothesis that difficulties using mental state information for social reasoning in individuals with ASD reflect an impairment in integrating conflicting information about mental states and outcomes – e.g., neutral intentions and negative outcomes – in moral judgment.

These behavioral results are consistent with previous neuroimaging study in which the neural activity in the right temporo-parietal junction, a region crucially involved in representing mental states, was transiently disrupted using transcranial magnetic stimulation during a task requiring intention attribution for moral judgment (Young et al., 2010). The authors showed that when the functional connectivity in this region is altered, the capacity to use information about a subject’s mental states (beliefs, intentions) is reduced in the case of accidental harm. It is likely that in circumstances of increased attentional and executive demands, ASD individuals may be impaired in the simultaneous maintenance and use of mental state information in working memory, rather than in inferring an agent’s mental states *per se*.

## A Deficit of the Episodic Control System

Studies suggest that these types of failures to integrate different types of information may reflect impairments in cognitive control in ASD (Geurts et al., 2009; Barbalat et al., 2014). Cognitive control refers to the processes that permit the flexible allocation of attentional and executive resources to guide thoughts and actions in the service of internal goals. Previous investigations have provided consistent evidence in favor of executive dysfunctions in autism (Hill, 2004; Hill and Bird, 2006; Geurts et al., 2009), although they have failed to demonstrate a specific profile that might differentiate autism from other psychopathologies. Here we explore several candidates for such specific dysfunctions.

Barbalat et al. (2014) investigated the hypothesis that executive dysfunction in autism affects the specific domain of episodic knowledge. The authors used an experimental paradigm which modeled three types of hierarchical organized executive control systems: sensory, contextual, and episodic, each recruiting distinct regions of the prefrontal cortex, along a rostro-caudal axis of the frontal lobe (Koechlin et al., 2003). While sensory control involves selecting the motor responses most appropriate to incoming stimuli, contextual control involves incoming signals that are congruent in time with the subject’s response, and episodic control supervises the retrieval of information stored in episodic memory into coherent, multimodal, episodic bindings. These bindings associate information related to incoming signals and specific action plans, and allow flexible switching between appropriate behaviors. Importantly, participants with ASD performed as well as comparison participants when they were required to select a specific response associated with sensory signals or the selection of a specific set of stimulus-response associations consistent with immediate contextual signals, but they demonstrated decreased accuracy when required to control information conveyed by episodic information (Barbalat et al., 2014). This result is in keeping with findings that individuals with ASD are impaired in the control of information conveyed by past events (Hare et al., 2007; Lind and Bowler, 2010). Interestingly, the more participants were impaired in episodic control processes, the more severe their symptoms were, as measured by the Autistic Spectrum Quotient (AQ).

In accordance with previous literature, this study shows that difficulties in ASD reflect the disruption of a specific functional system, the *Episodic Control system*, which encompasses the executive functions and episodic memory. Based on this finding, we hypothesize that in ASD, the disruption of this system compromises the ability to encode, integrate and consolidate episodic knowledge to be used for social and interpersonal reasoning. Episodic memory refers to the memory for personal experiences in specific temporal, spatial and situational contexts, integrated in a single representation. In humans, it is associated with a sense of self, subjective time, and self-awareness (Tulving, 2002).

Relevant to our proposal is the notion that the Episodic Control system allows episodic information from long-term memory to be temporarily represented and recalled as integrated, coherent, and multimodal in the Episodic Buffer (Baddeley, 2000). According to Baddeley (2000), the Episodic Buffer is a passive, multidimensional storage system capable of holding episodes. Crucially, it serves to create an interface between long-term memory and the visuo-spatial and phonological systems. Indeed, the episodic buffer is not itself responsible for the process of binding, but together with a central executive system, it is capable of holding episodes and integrated chunks of information. Thus, in carrying out this integration, the episodic buffer plays a key role in complex tasks, such as learning, comprehension and conscious reasoning, in both the intrapersonal and social domains.

Together with the episodic buffer, the episodic memory system plays a key role in facilitating social interaction and social understanding. Episodic memory is a source of memory for past social experiences specific to a time and place. But its main function is not to reminisce about the past, but to offer autobiographical, memory-based predictions for ongoing and distant future events by (1) providing spatially and temporally specific information about single experiences and (2) supporting the capacity to make novel inferences (Allen and Fortin, 2013). Episodic memory thus plays a crucial role in processing and using social information: the ability to navigate the social world depends on the capacity to remember specific experiences (e.g., who has been friendly and cooperated? who has been hostile and defected?). The importance of episodic memory in social functioning was demonstrated by Davidson et al. (2012), who investigated the quality of social relationships in three amnesic patients. The authors found that the patients had difficulty recalling details about places and specific episodes involving others, and also experienced problems using these memories. These difficulties hampered the patients’ flexible social functioning, severely disrupting their capacity to form and maintain close relationships.

Both children and adults with autism have difficulty recalling events and facts from their personal lives (Bruck et al., 2007; Goddard et al., 2007, 2014). According to Crane and Goddard (2008), this reduced ability to form episodic autobiographical memories reflects a deficit in using the self to organise memory retrieval. According to Powell and Jordan (1993), the development of personal episodic memories depends on the existence of an “experiencing self,” which encodes and stores events as part of a personal dimension. If the self is not constructed and involved as an agent in interaction with others, events are not experienced and encoded in a subjective and interpersonal way. Thus, “individuals with autism [are] aware of what [is] happening, but not aware that it [is] happening to them” (Powell and Jordan, 1993, p. 1). If individuals with autism have impairment in encoding, constructing and storing episodic autobiographical knowledge, they cannot benefit from past experience when processing both personal and social information. Thus, when they attempt to infer another’s mental state, they do not benefit from autobiographical and episodic memories in their social interactions. Individuals with ASD thus fail to fully develop a narrative self: a story about who they are based on their prior experiences and memories (Uddin, 2011).

Although the relationship between autobiographical memory and ToM has not been firmly established in typical populations, these two capacities do draw on a common network of brain activation, including the medial structures of the medial prefrontal cortex (PFC), anterior cingulate cortex, posterior cingulate cortex, precuneus, temporal poles, superior temporal sulcus and the hippocampus (Rabin et al., 2010). This overlap has been taken as evidence that individuals draw on a personal repertoire of past experiences to simulate or infer mental states in others (Buckner and Carroll, 2007; Hassabis and Maguire, 2007; Spreng et al., 2009).

The maintenance of integrated representations of prior knowledge about social events, agents, and specific memories of these agents and events are all necessary for successful mental state inference and social interaction. In typical development, the encoding, maintenance and recall of these integrated representations are often active and effortful, recruiting prefrontal brain regions and requiring additional cognitive resources that are not necessary for memory of simple features (Prabhakaran et al., 2000). The hippocampus binds these single elements into cohesive memories of individual events (Baddeley et al., 2010), and the medial prefrontal cortex creates complex representations of event sequences and memorizes them as script knowledge or event models (Grafman, 1989; Sirigu et al., 1995, 1996; Zalla et al., 2001).

In conclusion, when these integrated representations can not be formed or recalled, there may be a direct impact on explicit ToM judgments. Specifically, ToM difficulties may be partly attributable to the unavailability of episodic information for mental state ascription. The effects of such an impairment in individuals with ASD are particularly manifest in everyday social situations, when stimuli from multiple sources of information must be processed in an integrated manner.

## Conclusion

In the present article, we aimed to provide an integrative account of social impairments in autism. Our account draws on capacities both specific to social interaction, especially ToM, as well as on general cognitive capacities. Traditional theory of mind tasks emphasize social reasoning in a static context, and yet, in advanced versions, make a variety of demands on general cognition. Tasks requiring dynamic processing of social information and rapid social decision-making pose challenges to individuals with autism, but can also reveal the ways in which they achieve partial “compensation,” adapting their existing capacities to the task at hand. Such is the case for the acquisition of social priors: individuals with autism appear able to achieve limited social learning within a fixed context. Yet integrating information acquired in different contexts into one coherent representation appears to be very difficult for individuals with autism, both when they are provided with that information linguistically, and when they receive information through visual or other sensory channels or recall it from memory. In the domain of moral judgment, individuals with autism struggle to piece together information about intention and outcome to form a coherent representation.

Deficits in the use of prior knowledge and integration of socially relevant information acquired from diverse channels are broadly consistent with impairment in the episodic control system. We have argued that this impairment hampers the consolidation and retrieval in long-term memory of key social information such as event schemas and prior knowledge about the social and personal domains. Without the ability to maintain, across contexts, complex and consistent representations in memory of people, their mental states, and their specific personal experiences, individuals with autism lack an integrated conception of others – as well as themselves – as coherent and predictable social actors in the world. This account poses questions for future research, which should investigate the disrupted mechanisms encompassing the encoding, consolidation and retrieval of relevant social information in light of a different landscape of autobiographical episodic memory. For example, if the episodic buffer is selectively impaired in ASD, we would expect to find functional abnormalities in regions underpinning its recruitment, including the fronto-temporal network and the hippocampus (Rudner et al., 2007), during the execution of ToM tasks involving the recall of social information about known others.

Even if speculative, our unified framework is a step forward in explaining the gulf between ASD adults’ abilities in passing basic ToM tasks and the persistent and pervasive social deficits they still face in everyday life. Our account is distinctive in that it makes explicit many of the enormous informational and cognitive demands – far beyond ToM – managed seamlessly by TD individuals in real-life social situations. In so doing, it demonstrates some of the many ways in which ASD individuals can struggle with these demands, and also highlights the challenges of developing interventions that would enable ASD individuals to meet them. For example, one such intervention should explore how the social priors found in TD individuals, such as a preference for reciprocating a partner’s prosocial actions, can be learned by individuals with ASD, and then generalized to social situations outside the immediate context in which they were learned.

## Author Contributions

JK wrote the “Introduction,” “Thirty Years of ToM Studies: From False Belief to the Advanced ToM Tasks,” and “Challenges Beyond Compensation Strategies: Introducing New Conceptual and Inferential Demands” sections. TZ wrote the “Social Priors in Autism: Intention Inference and Social Learning,” “The Integration Deficit Hypothesis,” and “A Deficit of the Episodic Control System” sections. Both authors contributed to the Conclusion and edited the manuscript. JK completed final revisions.

## Conflict of Interest Statement

The authors declare that the research was conducted in the absence of any commercial or financial relationships that could be construed as a potential conflict of interest.
